# RETRACTED: Branda et al. Zoonotic Paramyxoviruses: Evolution, Ecology, and Public Health Strategies in a Changing World. *Viruses* 2024, *16*, 1688

**DOI:** 10.3390/v17070992

**Published:** 2025-07-16

**Authors:** Francesco Branda, Grazia Pavia, Alessandra Ciccozzi, Angela Quirino, Nadia Marascio, Giovanni Matera, Chiara Romano, Chiara Locci, Ilenia Azzena, Noemi Pascale, Daria Sanna, Marco Casu, Giancarlo Ceccarelli, Massimo Ciccozzi, Fabio Scarpa

**Affiliations:** 1 Unit of Medical Statistics and Molecular Epidemiology, Università Campus Bio-Medico di Roma, 00128 Rome, Italy; chiara.romano@unicampus.it (C.R.); m.ciccozzi@unicampus.it (M.C.); 2 Unit of Clinical Microbiology, Department of Health Sciences, “Magna Græcia” University of Catanzaro—“Renato Dulbecco” Teaching Hospital, 88100 Catanzaro, Italy; graziapavia@unicz.it (G.P.); quirino@unicz.it (A.Q.); nmarascio@unicz.it (N.M.); mmatera@unicz.it (G.M.); 3 Department of Biomedical Sciences, University of Sassari, 07100 Sassari, Italy; aciccozzi@uniss.it (A.C.); c.locci3@phd.uniss.it (C.L.); darsanna@uniss.it (D.S.); 4 Department of Veterinary Medicine, University of Sassari, 07100 Sassari, Italy; iazzena@uniss.it (I.A.); npascale@uniss.it (N.P.); marcasu@uniss.it (M.C.); 5 Department of Chemical Physical Mathematical and Natural Sciences, University of Sassari, 07100 Sassari, Italy; 6 Department of Public Health and Infectious Diseases, University Hospital Policlinico Umberto I, Sapienza University of Rome, 00161 Rome, Italy; giancarlo.ceccarelli@uniroma1.it

The journal retracts the article “Zoonotic Paramyxoviruses: Evolution, Ecology, and Public Health Strategies in a Changing World” [1] cited above.

Following the article’s publication, concerns were brought to the attention of the Editorial Office regarding an overlap between this publication [1] and an earlier article [2] published by a different group of authors.

Adhering to our complaints procedure, an investigation was conducted by the Editorial Office and Editorial Board, which confirmed an overlap between the two publications [1,2], without appropriate acknowledgement or citation. As a result, the Editorial Board has decided to retract this article [1] as per MDPI’s retraction policy (MDPI |Research and Publication Ethics, https://www.mdpi.com/ethics#_bookmark30).

This retraction was approved by the Editor-in-Chief of the journal *Viruses*.

The authors did not provide a comment on the decision.

## References

[1] Branda F., Pavia G., Ciccozzi A., Quirino A., Marascio N., Matera G., Romano C., Locci C., Azzena I., Pascale N. (2024). RETRACTED: Zoonotic Paramyxoviruses: Evolution, Ecology, and Public Health Strategies in a Changing World. Viruses.

[2] Haas G.D., Lee B. (2023). Paramyxoviruses from bats: Changes in receptor specificity and their role in host adaptation. Curr. Opin. Virol..

